# Genome-wide identification of Tomato Golden 2-Like transcription factors and abiotic stress related members screening

**DOI:** 10.1186/s12870-022-03460-9

**Published:** 2022-02-23

**Authors:** Zi-yu Wang, Shuang Zhao, Jun-fang Liu, Hai-yan Zhao, Xu-ying Sun, Tai-ru Wu, Tong Pei, Yue Wang, Qi-feng Liu, Huan-huan Yang, He Zhang, Jing-bin Jiang, Jing-fu Li, Ting-ting Zhao, Xiang-yang Xu

**Affiliations:** Key Laboratory of Biology and Genetic Improvement of Horticultural Crops (Northeast Region), Ministry of Agriculture and Rural Affairs, College of Horticulture and Landscape Architecture, Northeast Agricultural University, No. 600, Changjiang Road, Heilongjiang Province 150030 Harbin, P.R. China

**Keywords:** Tomato, *G2-like* family, Bioinformatics analysis, Expression patterns, Abiotic stresses

## Abstract

**Background:**

Golden 2-Like (G2-like) transcription factors play an important role in plant development. However, the roles of these *G2-like* regulatory genes in response to abiotic stresses in tomato are not well understood.

**Results:**

In this study, we identified 66 putative *G2-like* genes in tomato (*Solanum lycopersicum)* and classified them into 5 groups (I to V) according to gene structure, motif composition and phylogenetic analysis. The *G2-like* genes were unevenly distributed across all 12 chromosomes. There were nine pairs of duplicated gene segments and four tandem duplicated *SlGlk* genes. Analysis of the cis-regulatory elements (CREs) showed that the promoter regions of *SlGlk*s contain many kinds of stress- and hormone-related CREs. Based on RNA-seq, *SlGlk*s were expressed in response to three abiotic stresses. Thirty-six differentially expressed *SlGlk*s were identified; these genes have multiple functions according to Gene Ontology (GO) analysis and are enriched mainly in the zeatin biosynthesis pathway. Further studies exhibited that silencing *SlGlk16* in tomato would reduce drought stress tolerance by earlier wilted, lower superoxide dismutase (SOD), peroxidase (POD) activities, less Pro contents and more MDA contents.

**Conclusions:**

Overall, the results of this study provide comprehensive information on G2-like transcription factors and *G2-like* genes that may be expressed in response to abiotic stresses.

**Supplementary Information:**

The online version contains supplementary material available at 10.1186/s12870-022-03460-9.

## Background

Transcription factors (TFs), also known as trans-acting factors, constitute a group of proteins that play important roles in gene regulation by activating or repressing the transcription of downstream target genes, consequently controlling many cellular activities during plant growth and development [1–3]. TFs interact with DNA promoters through DNA-binding protein domains (DBDs) [4]. Plant growth and productivity are under constant threat from environmental stimuli in the form of biotic and abiotic stresses, and TFs participate in activating or inhibiting transcription in response to these stresses [5–7]. To date, 60 different TF families have been identified in plants [8]. In this study, we investigated the Golden 2-like (also called G2-like or Glk) TF gene family in tomato.

*Golden 2 (G2)* was first identified in maize, and its nomenclature follows that of *golden 1*, the first golden-producing factor [9]. Researchers have subsequently proved that *G2* acts as a novel transcriptional regulator of cellular differentiation in maize leaves [10]. GLK proteins belong to the GARP superfamily of TFs [6], which includes G2 in maize, Arabidopsis RESPONSE REGULATOR-B (ARR-B) proteins and the PHOSPHATE STARVATION RESPONSE1 (PSR1) protein in Chlamydomonas [11, 12]. Most G2-like genes have two highly conserved domains: a DBD (containing a helix-loop-helix [HLH] motif) and a C-terminal GCT box [13]. The DBD sequence is highly conserved among members of the GARP superfamily [14], occurring in both green algae and land plants; however, the GCT box is found only in land plants and is specific to the *GLK* genes [15]. In many land plants, *GLK*s encode a pair of partially redundant nuclear TFs. GLK TFs are important for the expression of nuclear photosynthesis-related genes and for chloroplast development [16].

*GLK* genes have been identified in moss (*Physcomitrella patens*), Arabidopsis, rice (*Oryza sativa*), pepper (*Capsicum annuum*) and tomato to date [15, 17–19], and these genes play roles in many aspects of plant biology. One vitally important function of *GLK*s is the regulation of chloroplast development. Previous studies have shown that, compared the wild type, g2 mutants had smaller chloroplasts and reduced amounts of thylakoid lamellae [20]. *GLK*s are also related to cellular differentiation. Three types of chloroplasts are present in C4 plants: C4 bundle sheath (BS) cells, C4 and C3 M (mesophyll) cells [21]. In contrast, in C3 plants, two *GLK*s act redundantly in chloroplast development [18]. *GLK*s regulate chloroplast development not only in the leaves but also in the fruit; these TFs can influence fruit quality by altering the sugar and carotenoid contents, as shown by studies in Arabidopsis, pepper and tomato [17–19, 22]. In addition, overexpression of GLK TFs in Arabidopsis leads to photosynthesis in root chloroplasts [23]. *GLK*s also play important roles in disease defense, as has been shown mainly in Arabidopsis [24–26] in low temperature and drought stress conditions [27]; in tolerance to ozone [28]; in terms of nitrogen-use efficiency [29]; and in the regulation of leaf senescence [30]. Y Yuan, X Xu, Z Gong, Y Tang, M Wu, F Yan, X Zhang, Q Zhang, F Yang, X Hu, et al. [31] reported that overexpression of SlARF6A, which could physically bind the two TGTCTC motif in the SlGLK1 promoter, increases *SlGLK1* transcriptional levels, and leads to enhanced chlorophyll accumulation and strong photosynthesis in tomato plants, which may result in strong tolerances against abiotic stresses such as drought and heat. Furthermore, *GLK*s are involved in the mediation of ubiquitin signaling [32].

However, previous studies of G2-like TFs in tomato have focused mainly on chloroplast development. Little is known about the roles of G2-like TFs in the stress resistance of tomato. In this study, we identified 66 *G2-like* genes via comprehensive bioinformatics analysis including gene structure, exon-intron organization, motif composition, gene duplication, chromosome distribution, phylogenetic relationships and promoter cis-elements. The expression of *G2-like* genes in response to abiotic stress (cold, drought and salt) based on RNA-seq data was also examined. Further studies exhibited that silencing *SlGlk16* would reduce the drought stress resistance of tomato plants. We aimed to identify genes that are important to stress resistance and thus lay a theoretical foundation for resistance improvement in tomato by providing genetic resources to accelerate the process of tomato breeding.

## Results

### Identification and physical property analysis of putative G2-Like Proteins in Tomato

A total of 66 G2-like proteins (designated *SlGlk1* to *SlGlk66*) were identified using the bioinformatics approach. Their basic physical information is presented in Table S2. The predicted molecular weights ranged from 20316.24 Da (*SlGlk60*) to 77633.43 Da (*SlGlk63*). Consistent with the molecular weights, *SlGlk60* (174 aa) had the shortest sequence and *SlGlk63* (708 aa) had the longest. The isoelectric point varied from 4.83 (*SlGlk34*) to 10.07 (*SlGlk46*), and the predicted aliphatic indices varied from 55.85 (*SlGlk21*) to 90.43 (*SlGlk58*). The instability indices varied from 33.02 (*SlGlk8*) to 63.18 (*SlGlk61*), and the instability indices of *SlGlk8/9/22/30/43/62* were lower than 40, indicating that these proteins were relatively stable. The hydrophobicity indices ranged from -1.003 (*SlGlk28*) to -0.29 (*SlGlk14*).

## Sequence alignment and phylogenetic analysis

**Fig. 1:**
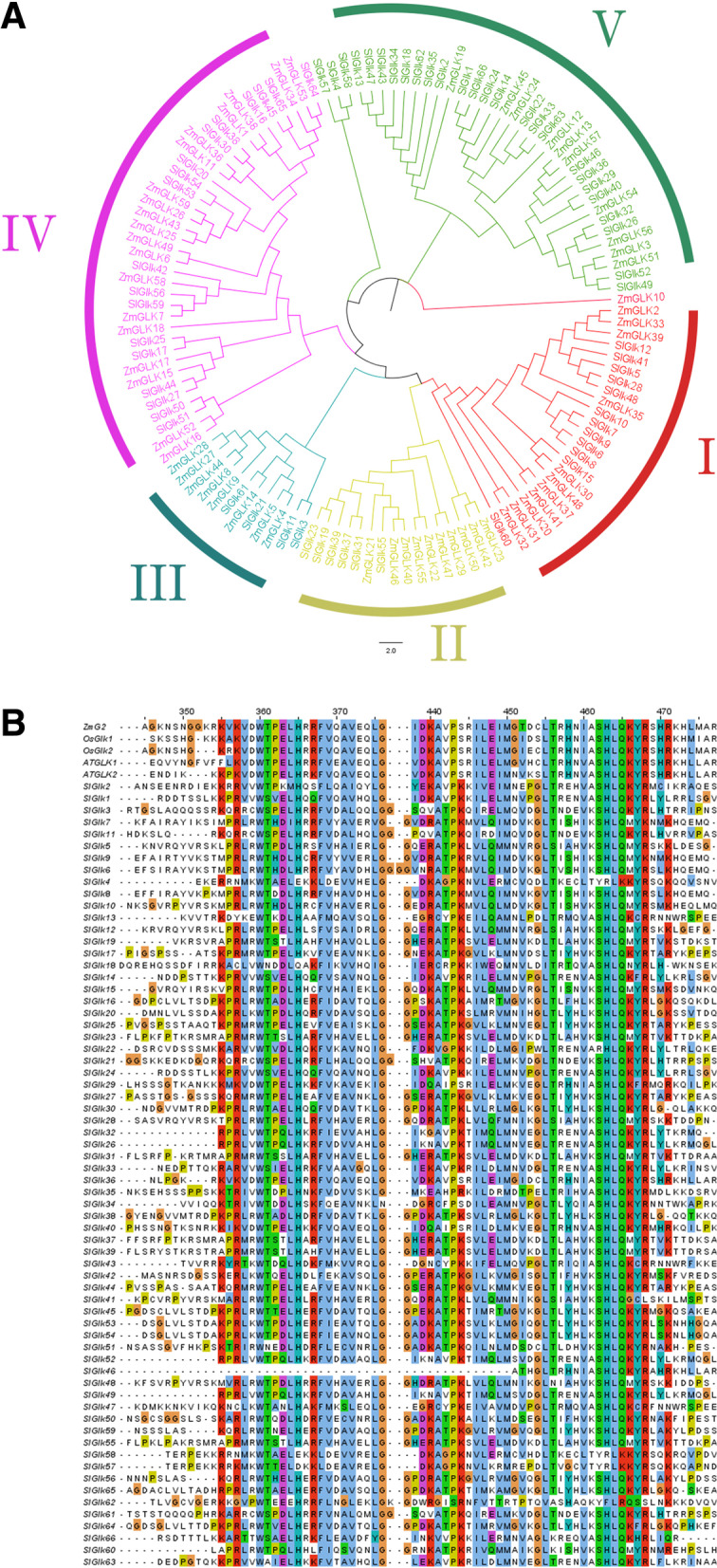
Sequence alignment and phylogenetic analysis of the G2-like proteins of maize and tomato. (**A**) Phylogenetic tree of ***ZmG***s and ***SlGlk***s. Each color represents one group, and five groups were found in total. (**B**) Alignment of the amino acid sequences of 66 putative G2-like in tomato, rice OsGLK1-2, Arabidopsis AtGLK1-2 and maize ZmG2. The different colors represent the different amino acids

The phylogenetic relationships of the G2-like proteins of tomato were classified into five groups according to their groupings with maize G2-like proteins and motif analysis of tomato G2-like proteins (Text S1 and Text S2). Groups I to V contained 12, 6, 4, 18 and 26 G2-like proteins, respectively (Fig. 1 A). Multiple sequence alignments (Fig. 1B) revealed that the genes were conserved across at least two regions of the HLH structure of the Myb-like domain: the first helix contained the initial sequence PELHRR (except in *SlGlk46*), and the second helix contained NI/VASHLQ. In addition, most *SlGlk*s in group IV had a specialized conserved Myb-CC-LHEQLE domain. A separate multiple sequence analysis was also performed in each subgroup (Figure S1). In the members of groups I and II, the form was mostly LHR/L/H/A; PELHRR and PDLHRR were the two types of conserved domains in group III members; the form of PELHRR was XD/ELHD/EX in group IV members; and XQ/ELHXX was the main type in group V members. The VI/NASHLQ domain was more conserved than the PELHRR domain was in all the groups. Four main types were present in members of all the groups: LKSHLQ, VKSHLQ, IKSHLQ and VASHLQ. On the basis of the results of the analysis of each group, we summarized the types of G2-like conserved domains (Table S3).

### Motif Analysis and Gene Structure of Tomato *G2-like* Genes

**Fig. 2:**
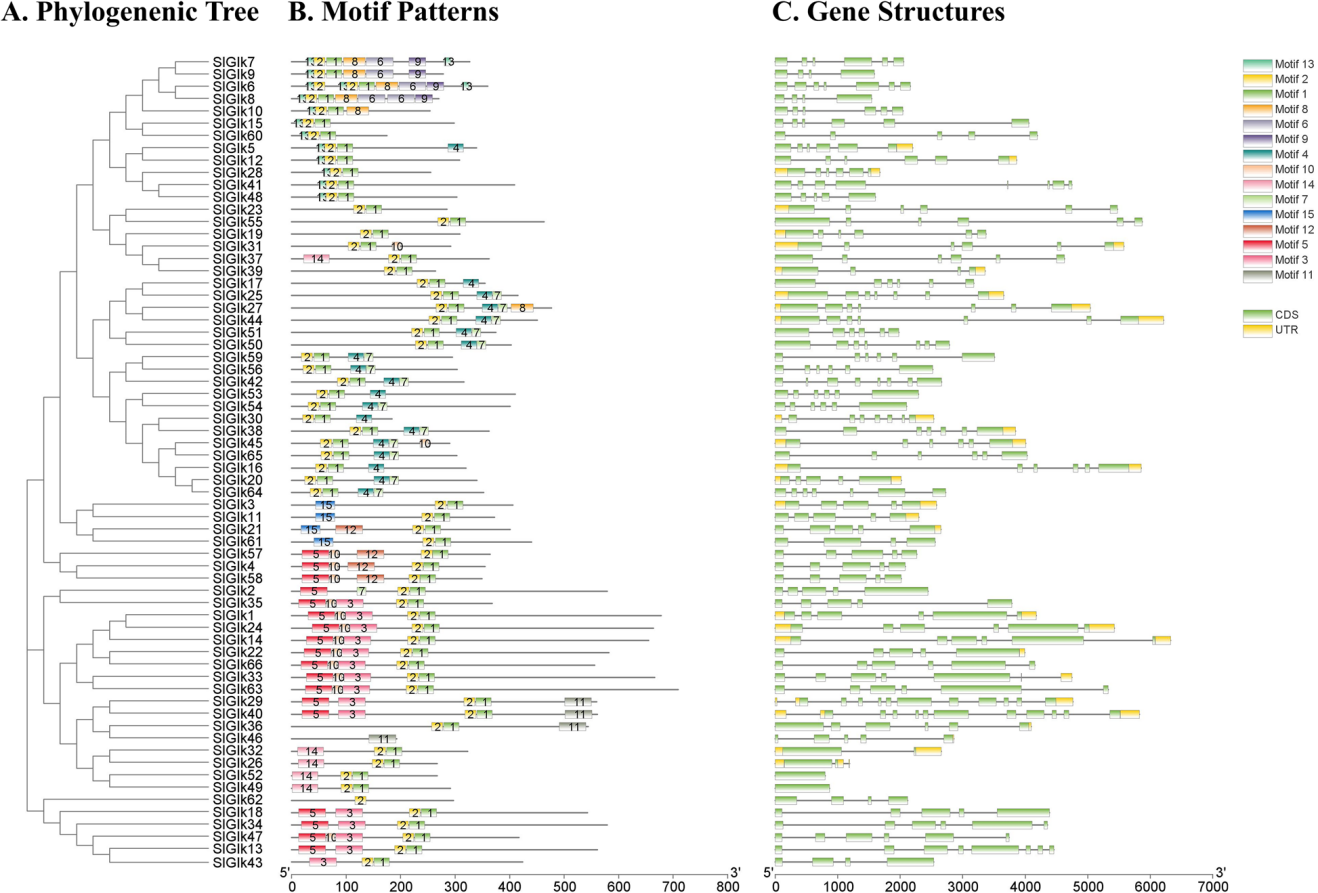
Phylogenetic tree, conserved protein motif structures and analysis of G2-like TFs-encoding genes from tomato. (**A**) The phylogenetic tree was constructed by MEGA 7.0 software using G2-like protein sequences. (**B**) Motif positions of tomato G2-like proteins. Fifteen motifs are displayed in boxes of different colors. (**C**) Intron and exon analysis of 66 predicted tomato G2-like genes. The green and yellow boxes represent exon regions and untranslated regions (UTRs), respectively; black lines indicate introns. The scales at the bottom are shown for measuring the lengths of the motifs, exons and introns

Fifteen putative motifs whose lengths varied from 13 to 50 amino acids were identified via the MEME website (Table S4) as representing the structure of all G2-like proteins. The results of the motif analysis are shown in Fig. 2B. Each protein sequence includes a different number of motifs (1~7), and each motif is present only once, with the exceptions of motif 13/motif 2 and motif 6, which were present twice in SlGlk6 and SlGlk8, respectively. Most G2-like proteins (83.33%) contained motif 1 and motif 2, which corresponded to the myb SHAQKYF domain based on Pfam database and Conserved Domain Database (CDD). We also found that the PLN03162 superfamily domain also contained motif 1 and motif 2. SlGlk62 and SlGlk46, which corresponded to the myb SHAQKYF domain and PLN03162 superfamily domain, contained only motif 2 and motif 11, respectively. The same motif compositions correspond to different domains, or different motif compositions correspond to the same domain, suggesting functional diversity. Details of the CDD results are showed in Table S5. The G2-like proteins clustered in the same group of the phylogenetic tree contained a similar motif composition besides SlGlk46/62, showing that they were highly conserved. For example, motif 15 was present only in group III members, which had very similar sequences. SlGlk6/7/8/9, which were formed from tandem duplications, also presented similar motif compositions. The function of the majority of these motifs needs to be further studied.

Studying the introns and exons of tomato, which were determined by the alignment of *G2-like* genes, would give more insight into the evolution of the G2-like family members in tomato. Intron and exon predictions are shown in Fig. 2 C, and the sequences of the *G2-like* genes are shown in Text S1. The number of exons varied from 1 to 11. More than half of the *G2-like* genes (62, 93.94%) had four or more exons, and only 4 genes (6.06%) had three or fewer exons. The conserved regions of all of the tandemly duplicated genes and segmentally duplicated genes presented similar exon distributions. Overall, the phylogenetic analysis results, motif composition and similar gene structure of the G2-like members in the same group provided reliable results for group classification.

### Chromosomal Location and Gene Duplication Events of *G2-like* Genes in Tomato

**Fig. 3:**
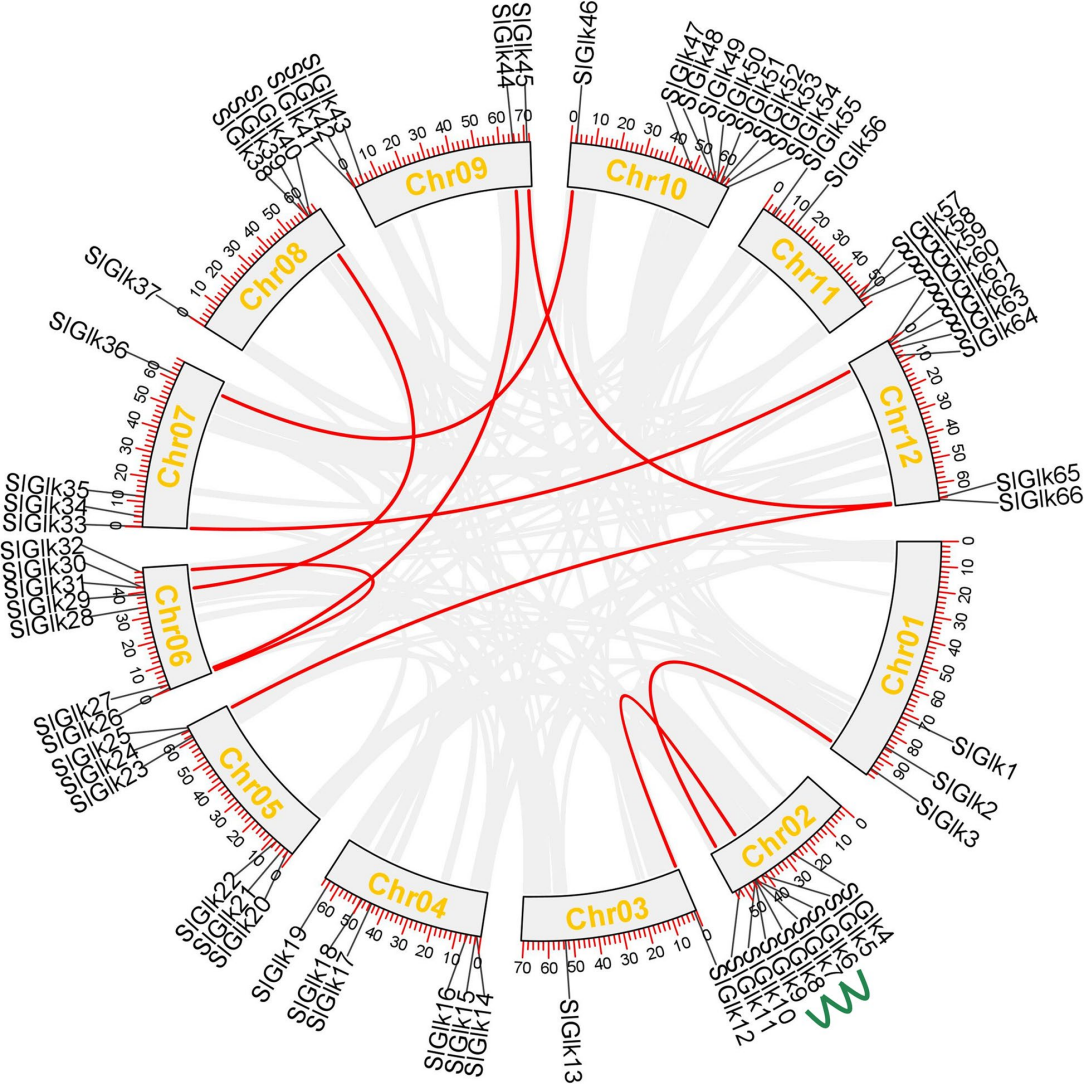
Chromosome distribution and duplication events of tomato G2-like genes. The different colored lines indicate different duplication events. The red lines indicate segmentally duplicated G2-like gene pairs, and the green lines indicate tandemly duplicated genes. The gray lines indicate all synteny blocks in the tomato genome. The lengths of the chromosomes can be estimated according to the scale provided

*SlGlk*s were unevenly distributed across the 12 tomato chromosomes, and the locations of most *SlGlk*s were on the proximal or distal ends of the tomato chromosomes. The number of *SlGlk*s per chromosome ranged from 2 to 9 (Chr10 had 9 genes; Chr03 only had 2 genes). As shown in Fig. 3, Chr01, which is the longest chromosome, contained only 3 genes. There was no significant positive correlation between chromosome length and gene number.

Genome duplication events, which are usually divided into three types (tandem duplications, segmental duplications and transposition events), occurred during plant evolution [33–35]. Tandem duplications are defined as chromosomal regions that are less than 200 kb in length and contain two or more genes [36]. There were 4 genes (*SlGlk6/7/8/9*) located on Chr02, which resulted from tandem duplications, that formed 1 tandem duplication event region. Using BLASTP and MCScanX, we also found 9 segmental duplications (18 *G2-like* genes in total) events (Fig. 3 and Table S6). Taken together, these results showed that some *SlGlk*s may have arisen via gene duplication.

### Analysis of CREs

**Fig. 4:**
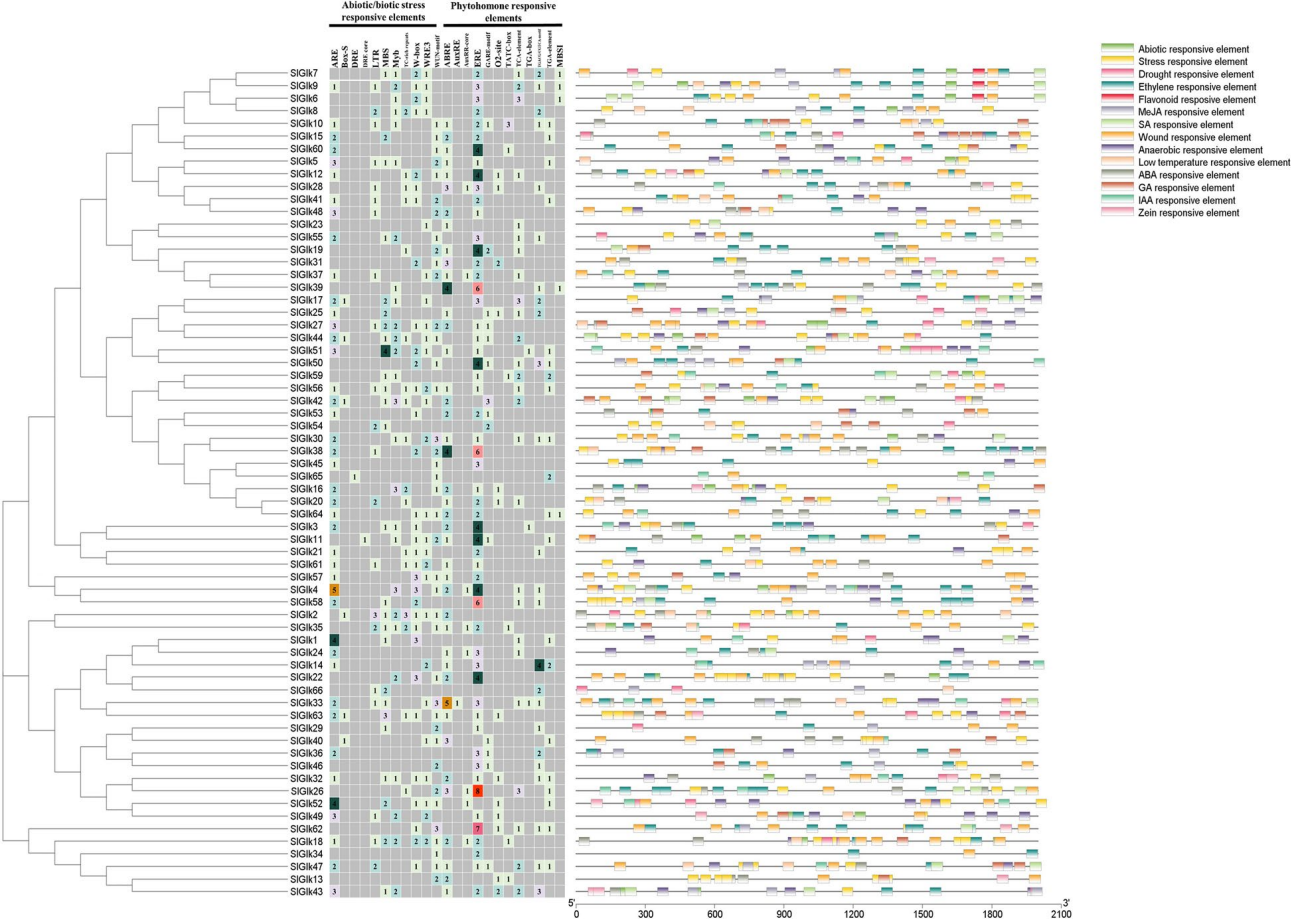
Analysis of CREs in promoter regions of *SlGlk*s according to information in the Plantcare database. Left: numbers in different colors indicated the numbers of the main cis-acting elements of *SlGlKs*. Right: fifteen CREs were predicted, each of which is displayed in a different color

To further determine the potential function of *G2-like* genes in response to abiotic stress, the CREs within the sequences 2 kb upstream from the translation start site of the *G2-like* genes were searched within the PlantCare database. Analysis of the promoters of *SlGlk*s in tomato revealed that all family members contained light-responsive elements and two core elements-the CAAT box and TATA box. Detailed elements are displayed in Table S7 and Fig. 4. There are also significant differences in the number of CREs among the promoters of the different members of the *G2-like* gene family. As shown in Fig. 4, the promoters of *SlGlk18* contained the most kinds of CREs (13), while *SlGlk34* contained only three kinds of CREs. Only 20 *SlGlk*s did not have any abiotic stress response elements, while the other *SlGlk*s contained at least one abiotic stress element, which indicated that the expression of more than half of the *G2-like* genes was related to abiotic stress. In addition, we found that 52 *SlGlks* (78.79%) had two or more hormone induction elements and that *SlGlk47* contained all five hormone induction elements, such as ABA-, IAA-, GA-, JA- and SA-induction elements. Regardless, analysis of the CREs showed that the number and distribution of CREs and that CREs in the same subgroup were not similar, which indicated that each *SlGlk*s are regulated by different combinations of TFs and that the expression of *SlGlk*s could be induced in response to different hormones and abiotic stresses.

### *G2-like* Gene Expression Patterns in Response to Different Abiotic Treatments

**Fig. 5:**
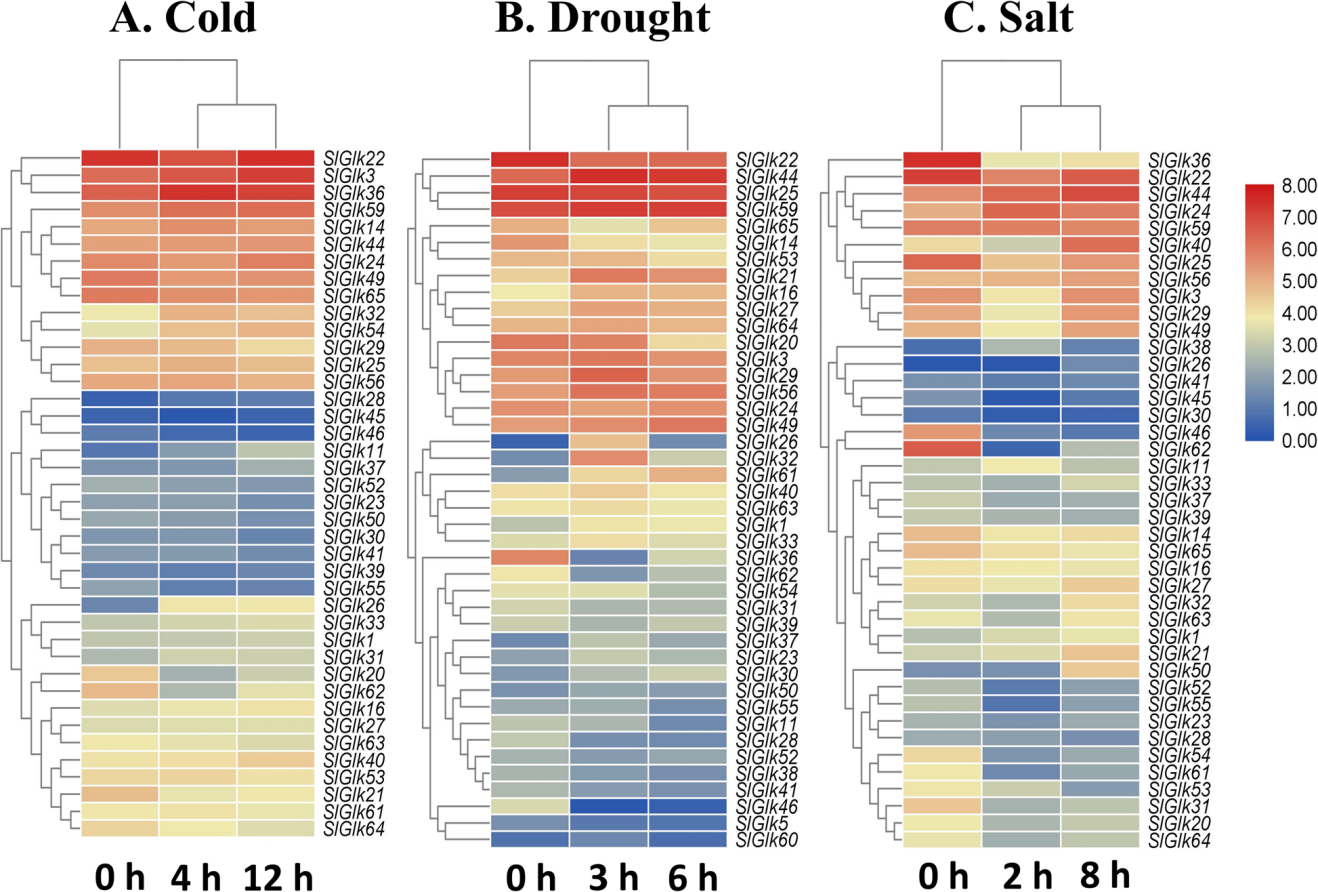
Expression profiles of *SlGlk* genes in response to three different abiotic stresses. Hierarchical clustering of the expression profiles of *SlGlk* genes in response to cold stress (**A**), drought stress (**B**) and salt stress (**C**)

**Fig. 6:**
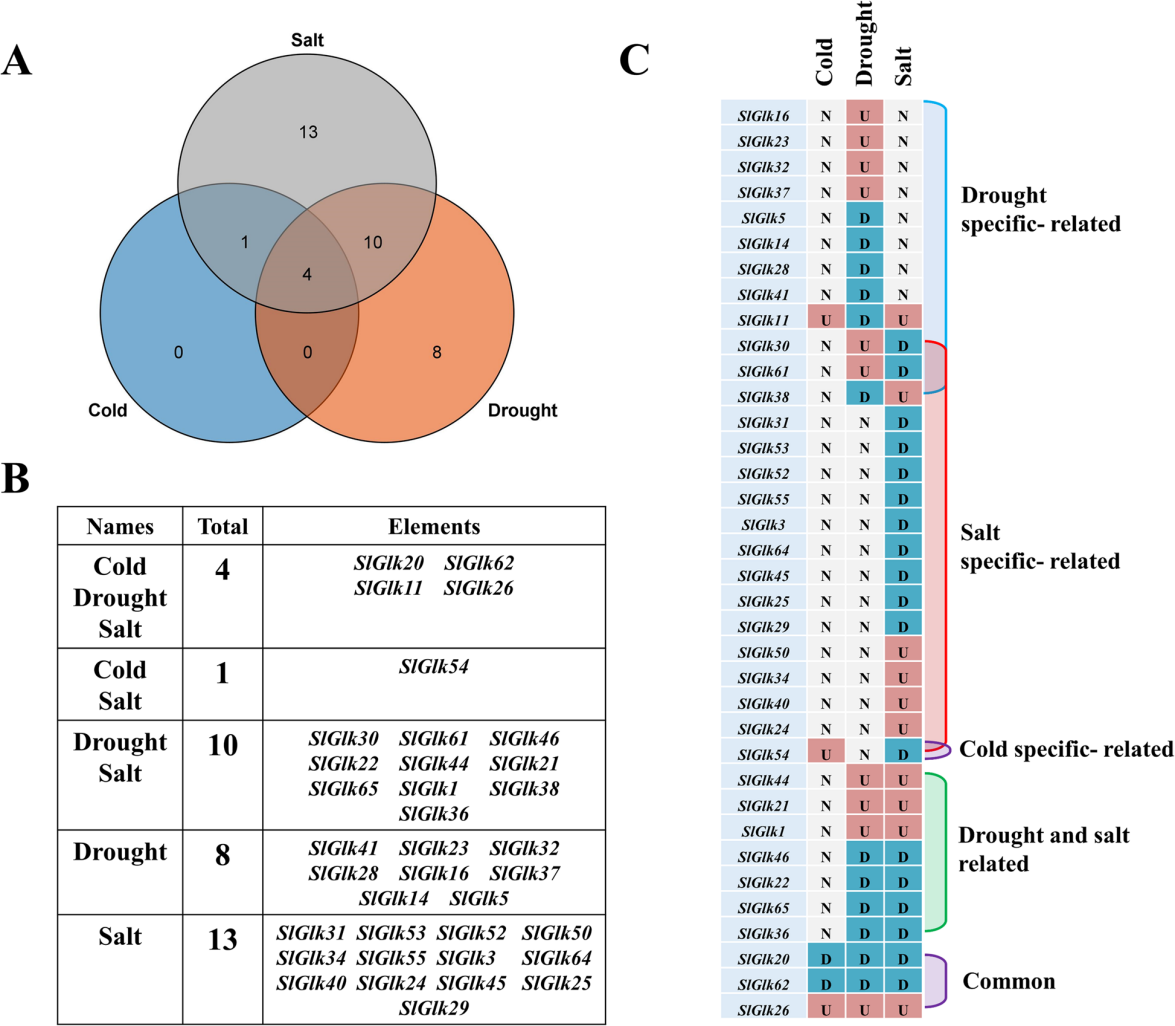
Different response patterns of DEGs in response to three abiotic stress treatments. (**A**) Venn diagram showing the overlap among genes differentially expressed in response to different abiotic stresses. (**B**) Detailed list of DEGs in the Veen diagram. (**C**) Representation of specifically expressed DEGs. U: Upregulated. D: Downregulated. N: No change

To explore the *G2-like* genes that respond to three different abiotic stresses, we downloaded RNA-seq data from the NCBI database (GSE148887, GSE148530 and GSE148353); data for *SlGlk4/9/43/57/58* were not found in the RNA-seq database. Nonetheless, the information was presented in the form of a heatmap (Table S8 and Fig. 5). As shown in Fig. 5, there were 40 genes expressed in response to cold stress, 42 genes expressed in response to drought stress and 41 genes expressed in response to salt stress. Among them, 20/19 genes, 17/25 genes and 16/25 genes were up/down-regulated by three abiotic stresses. We also found that the expression of *SlGlk22/44/25/59/29/56/24* was high in response to the three different stress treatments. These results indicated that some *G2-like* genes are involved in abiotic stress responses to help tomato seedlings adapt the nature environment. We defined *G2-like* genes as DEGs whose expression levels changed more than or equal to twofold than 0 h (and when P<0.05). A total of 53.03% of *G2-like* genes, including 5 cold stress-related genes, 22 drought stress-related genes and 27 salt stress-related genes, were differently expressed. As shown in Fig. 6 A, more than half of the DEGs (21 genes) responded to one stress treatment, and only 11 genes responded to two stresses. Four genes, *SlGlk11/20/26/62*, were expressed in response to all the stresses. The detailed gene list is shown in Fig. 6B. The DEGs that responded to only one abiotic stress or that differed from the other two genes, are shown in Fig. 6 C. The results showed that the expression of half of the drought specific-related genes was upregulated. In contrast, the expression of most salt specific-related genes was downregulated. Interestingly, we also found that the expression of *SlGlk44/21/1* and *SlGlk46/22/65/36* was up-/down-regulated under both drought and salt stresses respectively.

### Expression of Tomato *G2-like* Genes in Response to Abiotic Stress and Hormone Treatments

**Fig. 7:**
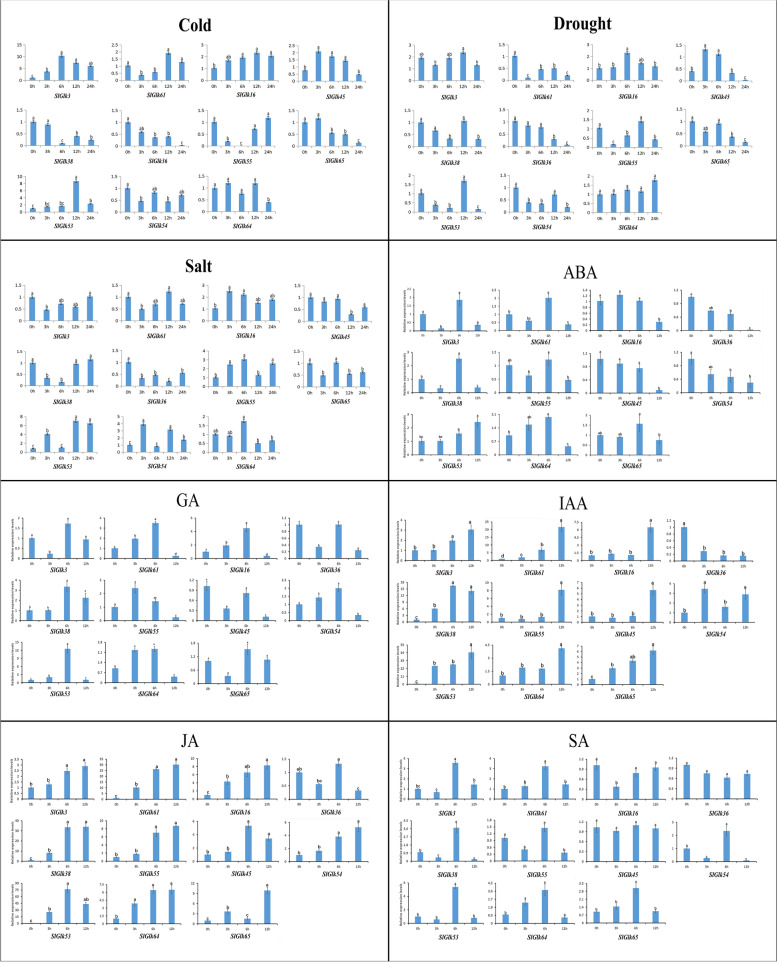
Expression patterns of *SlGlks* genes in response to abiotic stress and hormone treatments. The Y-axis indicates the relative expression level; the X-axis indicates hours of cold, salt, drought, ABA, GA, IAA, JA and SA treatments

To verified the expression data from RNA-seq and explore whether the expression of *SlGlk*s is affected by hormone treatments, we randomly selected 11 tomato *G2-like* genes from among the 36 DEGs to investigate through qRT-PCR the transcript levels of these 11 genes in response to different treatments. Detailed expression patterns of these *G2-like* genes are shown in Fig. 7. The expression of these 11 genes generally corresponded to RNA-seq results, indicating the RNA-seq results was reliable. We also found that the expression of these *SlGlk*s could be induced by at least 4 different abiotic stress and hormones treatments after 24 h and 12 h. Interestingly, we also found that the expression of some *SlGlk*s was induced/repressed by one treatment. For instance, the expression levels of all *SlGlks* except *SlGlk36* were upregulated under JA and IAA treatment, while the expression level of most *SlGlk*s were downregulated under drought and ABA treatment. The expression levels of seven *SlGlk*s (*SlGlk61/16/38/55/53/54/64)* were first upregulated but then were downregulated under GA treatment, and the expression of *SlGlk16* exhibited the same dynamic pattern under abiotic stresses. Several genes, such as *SlGlk38/55*, exhibited opposite expression patterns under different treatments. Taken together, these results showed that *SlGlk*s were influenced by most of the applied abiotic stress and hormone treatments.

### GO Enrichment and KEGG Enrichment Analysis of *G2-like* DEGs

**Fig. 8:**
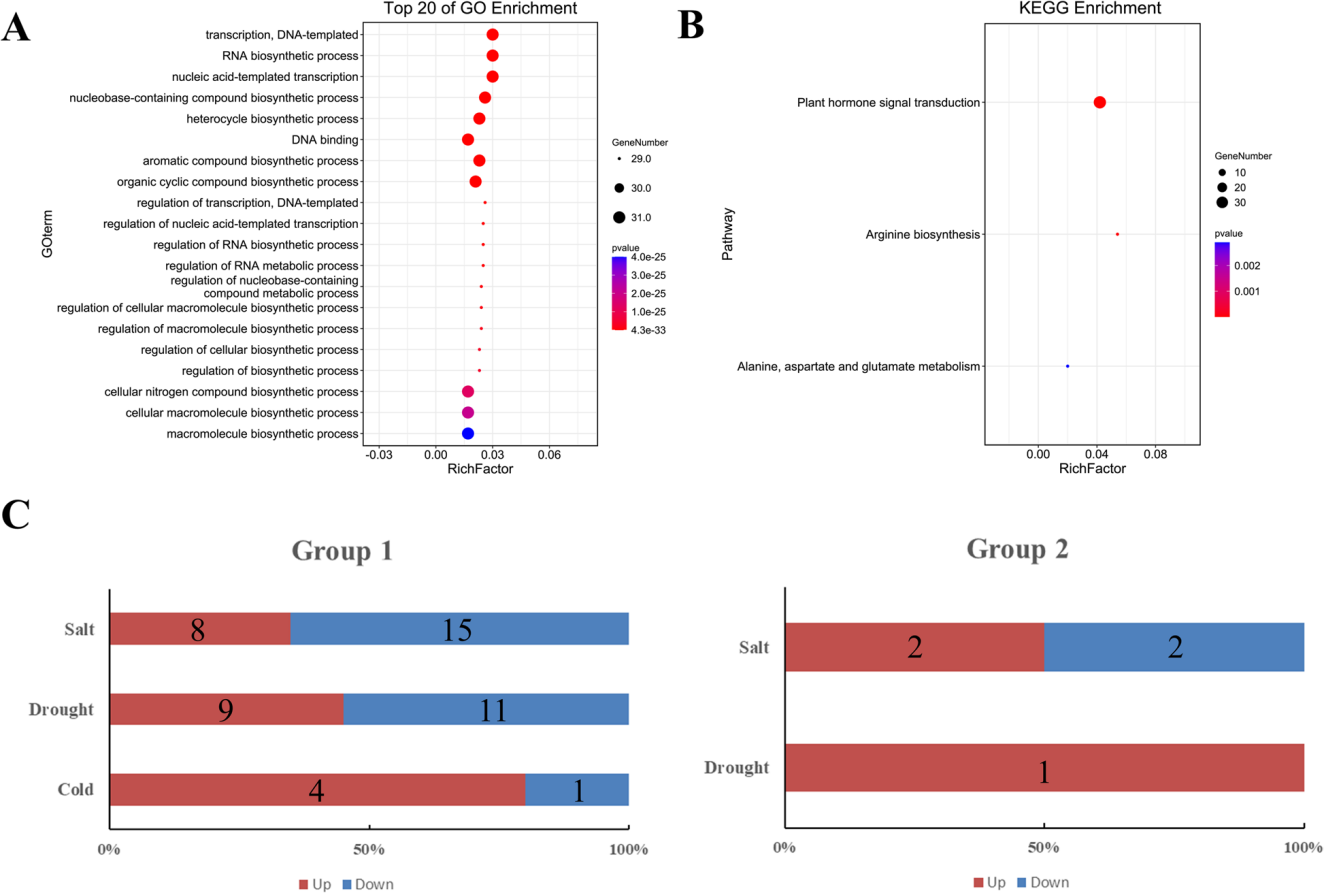
**A**. GO enrichment analysis of DEGs identified from RNA-seq. **B**. KEGG pathway enrichment of DEGs identified from RNA-seq. **C**. Comparisons of genes whose expression was upregulated and genes whose expression was downregulated between the two groups

To further determine the function of the 36 DEGs, GO enrichment and KEGG enrichment analysis was performed. The results of GO analysis can be generally divided into three categories: BP, CC and MF. We found that 87 GO terms were enriched for 31 DEGs, excluding *SlGlk22/23/38/14/34* and the detailed information was displayed in Figure S2. Biological regulation and cellular process were dominant in the BP category. CC clusters contained two subcategories, cell and organelle, with 31 DEGs, and binding was the only subcategory among the MF category. The top 20 terms for enrichment analysis with the whole genome as the background according to *P* value are shown in Fig. 8 A. With the exception of DNA binding, the remaining of 19 terms belonged to BP groups. Thirty-five DEGs (*SlGlk62* was excluded) were mapped to 3 KEGG pathways (*P* value<0.05): plant hormone signal transduction; arginine biosynthesis; and alanine, aspartate and glutamate metabolism (Fig. 8B and Table S9). Therefore, we divided the annotated DEGs into two groups: 31 DEGs in the plant hormone signal transduction pathway were assigned to group 1 while 4 DEGs (*SlGlk25/44/45/50*) related to amino acid metabolism were assigned to group 2. We further statistically analyzed the expression patterns of these genes under each abiotic stress in the two groups. We found that the expression patterns of DEGs in the same group were different. In group 1, the proportion of genes whose expression was downregulated was higher than the genes whose expression upregulated under drought and salt stress treatment, while the number of genes whose expression was upregulated was far greater than the number of genes whose expression was downregulated under cold stress treatment. In group 2, the proportions of genes whose expression was downregulated and the genes whose expression was upregulated were equal, and the expression of only one gene was upregulated. However, the expression of none of them changed under cold stress.

### Silencing *SlGlk16* reduced drought tolerance in tomato plants

**Fig. 9:**
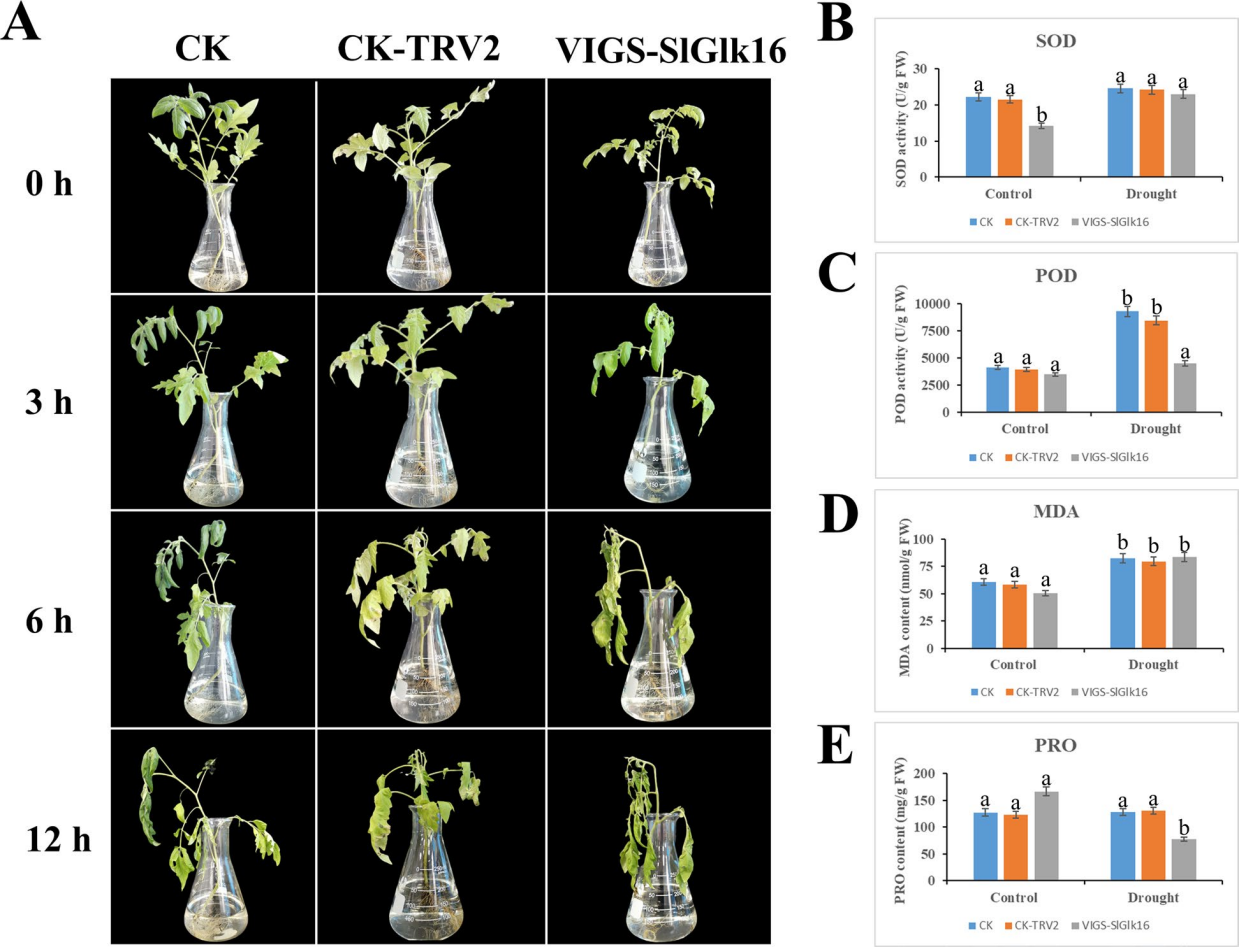
Silencing *SlGlk16* reduced drought stress tolerance in tomato plants. (**A**) Phenotypes of CK, CK-TRV2 and *SlGlk16*-silenced plants under drought stress. (**B**) SOD and (**C**) POD activities in CK, CK-TRV2 and *SlGlk16*-silenced plants under normal and drought stress condition. (**D**) MDA and (**E**) Pro contents in CK, CK-TRV2 and *SlGlk16*-silenced plants under normal and drought stress condition

Based on the previous study (the *SlGlks* expression under different treatments and RNA-seq databases under abiotic treatments), *SlGlk16* which is defined as drought-specific related DEG was selected for further analysis under drought stress. Figure S3 showed the expression level of *SlGlk16* in the infected tomato seedlings and we chose the plants (4 seedlings) whose *SlGlk16* expression level was less 50% than CK for further test. As shown in Fig. 9 A, the growth status of all plants was in similar before abiotic treatment, and the wilting of all plants was increased with increasing drought treatment time. Noteworthy, the leaves of *SlGlk16*-silenced plants began to wilt and the stem began to curl after 3 h drought treatment, while other plants only exhibited a slight curled stem. After 12 h later, the leave of *SlGlk16*-silenced plants showed wilting and drying more severe than the other two groups, although the above phenomena increased in severity in all the plants. The activity of SOD, POD and the content of MDA and Pro were measured for all plants under normal and drought treatment to explain the decreased drought tolerance in *SlGlk16*-silenced plants. After drought treatment, the activity of SOD and POD and the content of MDA increased in all plants. Under drought treatment, the activities of SOD and POD in *SlGlk16*-silenced plants were lower than that in the other two groups (Fig. 9B, C). However, the MDA contents in *SlGlk16*-silenced plants were higher than others after drought treatment (Fig. 9D). Interestingly, we also found that the Pro contents in detached leaves of *SlGlk16*-silenced plants were decreased after drought treatment and lower than CK and CK-TRV2.

## Discussion

In this study, 66 nonredundant *G2-like* genes, which were designated *SlGlk1* to *SlGlk66* on the basis of their chromosomal location, were identified and divided into five groups (I to V) according to their grouping with the sequences of those of Arabidopsis and maize. A previous study revealed that there are 59 and 45 Glk proteins in maize and sorghum, respectively, and the maize Glk proteins could be divided into seven groups [27]. We first obtained seven subgroups by phylogenetic analysis with maize Glk proteins, and then three subgroups whose members had a similar motif distribution were merged into one subgroup. Thus, the tomato SlGlk proteins were ultimately divided into five subgroups. The *SlGlk*s belonging to the same subgroups showed strong resemblance in terms of their structure and number of exons and introns, and they were adjacent in the phylogenetic tree. These consistent results suggested that the *SlGlk*s groupings were relatively reliable.

We aligned all 66 G2-like, AtGLK1/2, OsGLK1/2 and ZmGLK2 protein sequences via Jalview. In Fig. 1B shows that the SlGlks were conserved to a certain extent. We then aligned the 66 sequences according to their different groups. With respect to the PELHRR conserved domain, we observed that L and H were highly conserved in all the groups, and the PELHRR domain was present in various types. In addition, SHLQ was very conserved in the VI/NASHLQ domain, existing as only 8 variants, as shown in Table S3. These results suggested that the PELHRR domain may play a role in functional differentiation. The PELHRR and VI/NASHLQ domains were derived on the basis of an analysis of maize G2-like (ZmGlk) proteins, and we observed that these two conserved domains in SlGlks were similar to those in ZmGlks, but they also differed in their own composition [27]. Therefore, we deduced that even if the proteins from different species represented the same type of TF, their structure probably varied. The members of groups IV and V varied more, and hence, those members may be involved in more biological functions than those of the other groups.

According to the chromosomal location map, *SlGlk*s are widely and unevenly distributed across all 12 chromosomes of tomato, which could be due to insertions, deletions, duplications, and inversions. Gene duplication is a significant means for gene family expansion during the evolution of plant genomes. Among the *SlGlk*s, 12 duplication events have occurred: 9 segmental duplications and 3 tandem duplications (Fig. 3). Segmental duplications were obviously the main way in which the *SlGlk* gene family expanded in tomato. Many studies have shown that segmental duplication is more common than tandem duplication [27, 37–39], so the former may play an important role in the long-term evolutionary process. Some researchers believe that segmental duplication occurs regularly in slowly evolving gene families [27]. Taken together, these results indicated that tomato *G2-like* genes have probably been relatively conserved throughout the evolutionary process.

Many adverse conditions such as low temperature, salinity and drought, act as limiting factors in the process of plant growth and development. Many studies have also indicated that hormones are necessary for plant responses to biotic and abiotic stresses [40–43]. Hence, we analyzed the expression of 11 select tomato *G2-like* genes under three abiotic stresses and five hormone treatments. Previous studies have shown that orthologous genes in different species have conserved ancestral gene functions, but paralogous genes have distinct functions due to gene duplication [44, 45]. Interestingly, in a previous study, we found that the expression of *SlGlk38* and its ortholog in maize, *ZmGlk11*, exhibited opposite patterns in response to cold and drought stresses [27], suggesting that *SlGlk*s may have lost or gained new functions during evolution. In the present study, the expression of *SlGlk45* was induced under IAA and JA treatment and repressed under cold, salt, drought, ABA and GA treatment. However, the expression profiles of *SlGlk65* differed from those of *SlGlk45*, although both segmentally duplicated genes (Fig. 7). The expression of most the selected genes was in response to IAA and JA. Studies have also shown that *AtGlk1* is related to SA and JA signaling in disease defense, including that against *Hyaloperonospora arabidopsis* (Hpa) Noco2 and *Botrytis cinerea* [46–51]. In addition, *OsGlk1* may participate in resistance to pathogen invasion [48], and *AtGlks* may be useful in tolerance to Cucumber mosaic virus [24]. Therefore, we conclude that *SlGlk*s may play important roles in disease resistance. Further study of these genes might allow us to obtain more stress-resistant and disease-resistant tomato varieties.

It has been reported that TFs can integrate multiple CREs to regulate gene expression [52]. In the present study, CRE analysis showed that the promoter region of *SlGlk*s have many abiotic- and hormone-related elements, such as MBS, ABRE, GARE-motif and LTR. Interestingly, we found that the expression of *SlGlk*s is not strongly correlated with CREs. For example, we found that *SlGlk3/64/65* contain IAA-esponsive elements, so we surmised that the expression of *SlGlk3/64/65* could be induced by IAA treatment, which is consistent with our qPCR findings. Conversely, the promoters of *SlGlk16/38/45/61/53*, which contain no IAA-responsive elements, could also be induced by IAA treatment (Figs. 4 and 7). Thus, we speculated that CREs with unknown functions are related to IAA. We also found that different numbers of the same CRE would affect the expression level of *SlGlk*s. For example, the expression level of *SlGlk53* was more significantly induced than that of *SlGlk16* was under GA treatment; the promoter region of *SlGlk53* contained three GA-responsive elements, whereas that of *SlGlk16* contained only one (Figs. 4 and 7).

On the basis of RNA-seq data, the number of DEGs and specific responsive of *SlGlk*s was not vastly different under drought and salt stresses, while the fewest number of genes were differentially expressed in response to cold stress. These results indicated that *G2-like* genes play an important role in drought and salt responses. Thirty-five DEGs were enriched in KO04075 which is related to zeatin biosynthesis. Zeatin is a plant growth regulator, which can promote cell growths, prevent chlorophyll and protein degradation, maintain cell vitality and delay plant senescence. Thus, we suspected that *G2-like* genes could regulate the content of zeatin in plants, so that the tomato plants could adapt to the abiotic stresses.

By comparison with the well-studied function of G2-like TFs in chloroplast development and biotic stress [22, 24], there is little known about their role in abiotic stresses. To compensate this defect, we used VIGS to initially assess the potential involvement of *SlGlk16* in abiotic stress. In the present study, we found that silencing *SlGlk16* in tomato exhibited reduced drought stress tolerance by earlier wilting and variation of corresponding physiological indexes under drought stress, which indicated that *SlGlk16* is a positive regulator in drought stress. It is known that the activities of SOD and POD play an important role to protect cell membranes from ROS attack [53]. The contents of MDA and Pro could reflect the damage degree of cell and help plants adapt osmotic stress [54, 55]. In our study, we found that the activities of SOD and POD and Pro contents in *SlGlk16*-silenced plants were lower than the other two groups under drought stress while the *SlGlk16*-silenced plants had higher MDA contents, which suggesting that silencing *SlGlk16* would reduce the stability of cell membrane and the activity of peroxidases and finally reduced their drought tolerance.

## Conclusions

In this study, 66 *G2-like* genes in the tomato genome were characterized and classified into five groups with distinct structures and motif compositions. These genes were unevenly distributed across the 12 tomato chromosomes, and 21 *SlGlk*s were considered duplicate genes. qRT-PCR and RNA-seq data showed that most G2-like genes could respond to different abiotic stress and hormone treatments, and KEGG analysis revealed that G2-like proteins may be related to zeatin biosynthesis. Finally, *SlGlk16* was defined as a positive regulator under drought stress. Taken together, these results provide comprehensive information to further study the function of *G2-like* gene family members in response to abiotic and biotic stresses.

## Methods

### Identification of *G2-like* Genes and Construction of Phylogenetic Trees

The tomato sequence file SL3.0 was downloaded from the Solanaceae Genomics Network (SGN) (http://solgenomics.net). The reported G2-like protein sequences of *Arabidopsis* (AT2G20570.2 and AT5G44190.1) from The Arabidopsis Information Resource (TAIR 9) (https://www.arabidopsis.org/), *Oryza sativa* (LOC4340977 and LOC4326363), *Zea mays* (LOC542493) and *Nicotiana tabacum* (LOC107817975) were used as query sequences to identify G2-like sequences within tomato protein databases by BLASTP (E-value: 1e-5). To further test the obtained tomato sequences, we compared them with G2-like gene family classification criteria, in which the members of G2-like TFs should include G2-like domains and exclude Response reg domain according to the Plant Transcription Factor Database (Pln TFDB 3.0) (http://plntfdb.bio.uni-potsdam.de/v3.0/), via the HMMER program (http://www.ebi.ac.uk/Tools/hmmer/). We then used SMART (http://smart.embl-heidelberg.de/) to further verify the identities of all the proteins. Finally, the physical parameters of all the confirmed amino acid sequences were estimated using the online software ExPASy (http://web.expasy.org/protparam/). Protein and nucleic acid sequences of maize were acquired from a previous publication [27].

Multiple protein sequence alignments of tomato and maize were executed using ClustalX [56]. Afterward, an unrooted phylogenetic tree was constructed with MEGA 7.0 software [57] via the maximum likelihood method with the Jones-Taylor-Thornton (JTT) model and 1000 bootstrap replicates. G2-like proteins were classified according to the topology and bootstrap values of the phylogenetic tree. The G2-like protein sequences of tomato and maize are listed in Texts S1 and S2.

### Gene Structure Analysis, Motif Analysis and Cis-regulatory Element (CREs) Analysis, Chromosomal Location and Gene Duplication Events of *G2-like* Genes in Tomato

Gene structure analysis included the prediction of introns and exons, which were analyzed via SGN (http://solgenomics.net). The conserved motifs of the tomato G2-like proteins were evaluated via online MEME software (http://meme-suite.org/). The number of motifs found in MEME was limited to 15 and other paraments default, the positions of the conserved domains were predicted using Jalview software, the exon-intron structures were displayed by TBtools [58]. The CREs were selected from the 2000 bp upstream sequences of the start codon of all SlGlks via the PlantCARE database (http://bioinformatics.psb.ugent.be/webtools/plantcare/html/) [59].

Chromosomal position information was obtained from the SGN (http://solgenomics.net), and all *SlGlk*s were mapped onto the tomato chromosomes by Circos [60]. The Multiple Collinearity Scan Toolkit (MCScanX), with the default parameters, was used to determine the gene duplication landscape [61]. A syntonic map was subsequently displayed by TBtools [58].

### RNA-seq Analysis and Gene Expression Heatmap of *G2-like* Genes

The expression patterns of G2-like genes in response to cold, drought and salt stresses were revealed by analyzing the RNA-seq data. The original RNA-seq data were obtained from the NCBI database, of which the accession numbers with respect to the response to cold, drought and salt stresses were GSE148887, GSE148530 and GSE148353 respectively, and the RNA-seq data of Micro-Tom (CK) was only used. The fragments per kilobase per Million (FPKM)-normalized fragment data of significantly expressed genes (FPKM>1) was log_2_ transformed, and the expression patterns of G2-like genes were visualized by a heatmap via Multiple Experiment Viewer 4.0.

### Plant Material and Treatments

The tomato (*Solanum lycopersicum*) cultivar Micro-Tom and Money Maker (MM), which was provided by the Tomato Research Institute of Northeast Agricultural University, was used in this study. All seedlings were grown under a day: night temperature of 20~25℃: 13~15℃ at 45% relative humidity under a 13:11 h light: dark photoperiod. Five-week-old Micro Tom plants were irrigated with 15% polyethylene glycol (PEG) 6000 and 200mM NaCl for drought and salt treatments, respectively; for cold treatment, tomato plants were placed in a 4℃ growth chamber. Leaf samples from plants subjected to abiotic stress treatments were collected randomly after 0, 3, 6, 12 and 24 h of stress imposition. For phytohormone treatments, solution of 100 µM abscisic acid (ABA) and jasmonic acid (JA), 400 µM salicylic acid (SA), 450 mg/L indoleacetic acid (IAA) and 300 mg/L gibberellic acid (GA) were sprayed onto tomato plants in accordance which complied with the requirements. Leaf samples were collected randomly at 0, 3, 6, 12 h after the phytohormone treatments were applied. Each sample (containing three to four leaves) with three biological replicates were frozen at -80℃ for RNA isolation. Each replicate contained five seedlings.

### RNA Extraction, cDNA Synthesis and Quantitative Real Time PCR (qRT-PCR) Analysis

Total RNA was extracted from the samples using TRIzol. First-strand cDNA was synthesized using 1 µl of total RNA via a transcript kit purchased from Beijing TransGen Biotech. RT-qPCR was carried out using AceQ qPCR (SYBR Green Master Mix) in conjunction IQ5. The Actin gene (Solyc11g005330.1.1) was used as an internal control. For each qRT-PCR, 1 µl of diluted cDNA, 0.5 µl each of forward primer and reverse primer, 10 µl of SYBR and 8 µl of ddH_2_O were used in a 20 µl reaction. The reaction was carried out as follows: 95 °C for 5 min, followed by 40 cycles of 94 °C for 5 s, 60 °C for 15 s and 72 °C for 10 s; three biological replicates were included. The 2^−ΔΔCt^ method was used for quantification and Actin as a reference gene. Information about the primers is shown in Table S1. Three replicates were necessary for each time point.

### Gene Ontology (GO) and Kyoto Encyclopedia of Genes and Genomes (KEGG) Enrichment Analysis of Differentially Expressed Genes (DEGs) of G2-like TFs

GO enrichment analysis of DEGs was performed using the GOseq R package. The GO analysis involved three categories: molecular function (MF), biology progress (BP) and cellular component (CC). We inferred the behavior of cells or organisms on the basis of the genome or transcriptome of genes annotated to KEGG pathways [62], and KOBAS software was used to determine the DEGs enriched in the various KEGG pathways [63]. The results of the GO and KEGG analyses are shown via bubble chart.

### Generation of *SlGlk16*-silenced plants and phenotypic observation under drought stress

The *SlGlk16* fragment was amplified from cDNA made from MM RNA (leave samples) via specific primers (Table S1) designed in accordance with N Fernandez-Pozo, HG Rosli, GB Martin and LA Mueller [64]. The target sequence of *SlGlk16* was then cloned into tobacco rattle virus RNA2 (TRV2) via ClonExpress II One Step Cloning Kit (Vazyme, China). To obtained *SlGlk16*-silenced plants, the fusion plasmid TRV2-*SlGlk16* was introduced into *Agrobacterium tumefaciens* GV3101 which was then infiltrated into leaves of five fully expanded leaves of MM seedlings according to the method [65]. There were three experimental replicates, and each replicate involved ten MM seedlings. The expression levels of *SlGlk16* in infiltrated seedlings were determined by qRT-PCR, and the lines with the expression levels less than 50% than CK were used for further drought stress treatment. Each sample (containing two to three leaves) with three biological replicates.

For drought-resistance test, *SlGlk16*-silenced seedlings, CK and CK-TRV2 grown in Hoagland’s solution for 24 h and then transferred into 15% PEG6000 solution for 12 h. We observed the phenotype of the plants at 0, 3, 12 h after drought stress treatment.

## Physiological measurements

Leaf samples were collected randomly from CK, CK-TRV2 and *SlGlk16*-silenced seedlings before and after drought stress treatment. Each sample (containing two to three leaves) with three biological replicates. We used the corresponding kits (Keming, China) to measure the contents of proline (Pro), malondialdehyde (MDA) and determine the activities of superoxide dismutase (SOD) and peroxidase (POD) according to the manufacturer’s protocol. Three independent biological replicates were used for all measurements.

### Statistical analysis

Statistically significant differences between CK, CK-TRV2 and *SlGlk16*-silenced plants in terms of the measured parameters were tested by Ducan’s multiple range tests. Different lowercase letters indicate significant differences (P<0.05). Bars are SE (n=3).

## Supplementary Information

**Additional file 1.**

**Additional file 2.**

**Additional file 3.**

**Additional file 4.**

**Additional file 5.**

**Additional file 6.**

**Additional file 7.**

**Additional file 8.**

**Additional file 9.**

**Additional file 10.**

**Additional file 11.**

**Additional file 12.**

**Additional file 13.**

**Additional file 14.**

## Data Availability

The web link to the RNA-seq database are as follows: Comparative transcriptome analysis between two tomato materials under cold stress. https://www.ncbi.nlm.nih.gov/geo/query/acc.cgi?acc=GSE148887. Comparative transcriptome analysis between two tomato materials under drought stress. https://www.ncbi.nlm.nih.gov/geo/query/acc.cgi?acc=GSE148530. Comparative transcriptome analysis between two tomato materials under salt stress. https://www.ncbi.nlm.nih.gov/geo/query/acc.cgi?acc=GSE148353. We declare that the dataset(s) required to reproduce the results of this article are included in the article and additional file(s) available in the journal webpage.

## Abbreviations

G2-like: Golden 2-Like
TFs: Transcription factors
CDD: Conserved Domain Database
CREs: cis-regulatory elements
SGN: Solanaceae Genomics Network
TAIR: The Arabidopsis Information Resource
Pln TFDB 3.0: Plant Transcription Factor Database
JTT model: Jones-Taylor-Thornton model
MCScanX: Multiple Collinearity Scan Toolkit
FPKM: Fragments per kilobase per Million
MM: Money Maker
PEG: Polyethylene glycol
ABA: Abscisic acid
JA: Jasmonic acid
SA: Salicylic acid
IAA: Indoleacetic acid
GA: Gibberellic acid
qRT-PCR: Quantitative real
GO: Gene Ontology
KEGG: Kyoto Encyclopedia of Genes and Genomes
DEGs: Differentially Expressed Genes
ME: Molecular function
BP: Biology progress
CC: Cellular component
TRV2: Tobacco rattle virus RNA2
Pro: Proline
MDA: Malondialdehyde
SOD: Superoxide dismutase
POD: Peroxidas
