# Supplementary material for: Genome-wide identification of Tomato Golden 2-Like transcription factors and abiotic stress related members screening
Source: BMC Plant Biol. 2022 Feb 23;22:82. doi: 10.1186/s12870-022-03460-9 (PMC8864820; doi:10.1186/s12870-022-03460-9)
Supplement: Supplementary file 4 — Additional file 4. [file 12870_2022_3460_MOESM4_ESM.docx]

| Table S4 Information of motifs in tomato G2-like proteins | | |
| --- | --- | --- |
| Name | Patterns | Sequences |
| Motif 1 | 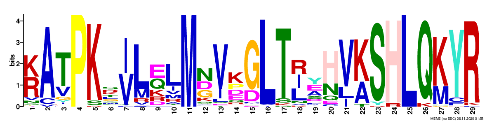 | KATPKSILZLMNVKGLTRYHVKSHLQKYR |
| Motif 2 | 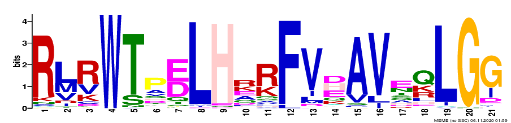 | RLRWTPELHRRFVDAVEQLGG |
| Motif 3 | 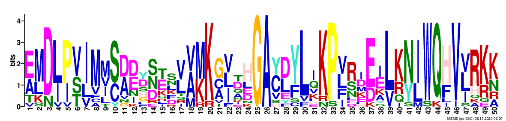 | EMDLPVIMMSDDYSESVVMKGVTHGAYDYLIKPVRDEELKNIWQHVVRKK |
| Motif 4 | 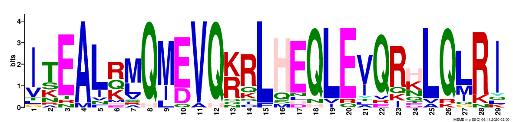 | ITEALRMQMEVQKRLHEQLEVQRHLQLRI |
| Motif 5 | 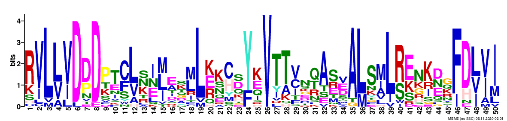 | RVLLVDDDPTCLKILEKMLKKCSYKVTTVNQASEALSMLRENKDGFDLVI |
| Motif 6 | 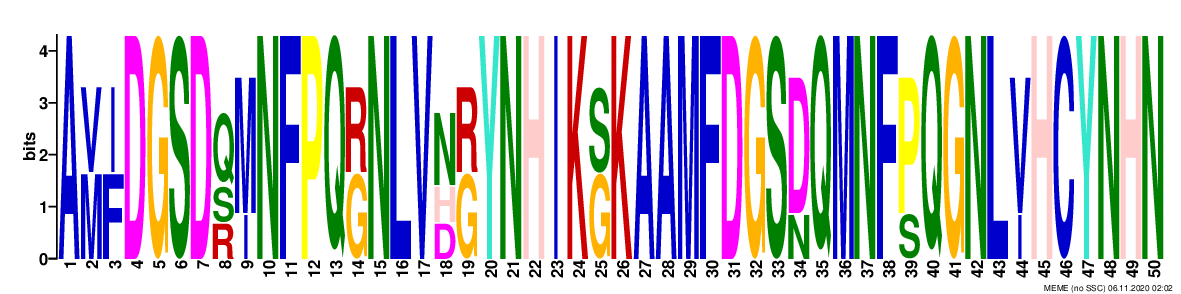 | AVFDGSDQMNFPQGNLVNGYNHIKGKAAMFDGSDQMNFPQGNLVHCYNHN |
| Motif 7 | 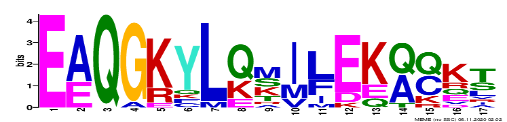 | EAQGKYLQMILEKQQKT |
| Motif 8 | 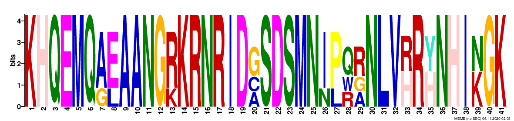 | KHQEMQAEAANGRKRNRIDGSDSMNIPQRNLVRRHNHIKGK |
| Motif 9 | 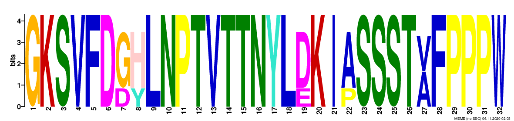 | GKSVFDGHLNPTVTTNYLDKIASSSTVFPPPW |
| Motif 10 | 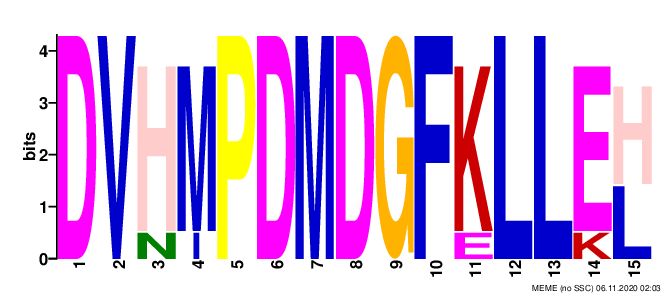 | DVHMPDMDGFKLLEH |
| Motif 11 | 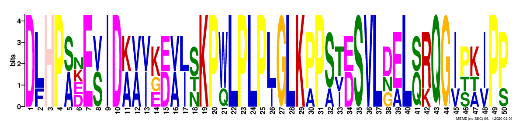 | DLHPADEVIDKVIKDVJSKPWLPLPLGLKPPSTDSVLDELQRQGIPKIPP |
| Motif 12 | 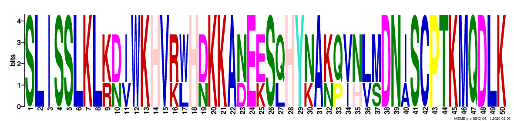 | SLISSLKLKDIWKHVRWHDKKANEESQHYNAKQVNLMDNISCPTKMQDLK |
| Motif 13 | 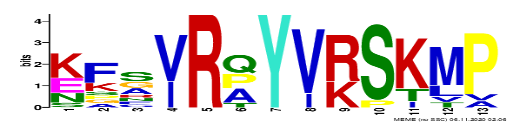 | KFSVRQYVRSKMP |
| Motif 14 | 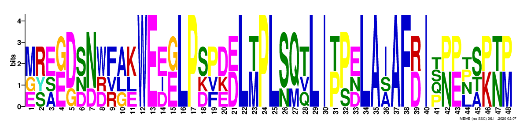 | MREGDSNWFAKWEEGLPSPDDLTPLSQTLIPPELAIAFDISPPPSPTP |
| Motif 15 | 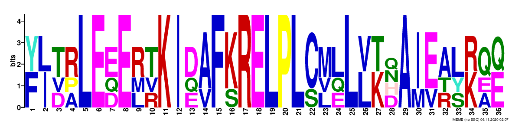 | YJTRLEEERTKIDAFKRELPLCMLLLKHAIEALKQZ |
