# Supplementary figures and images for: Genome-wide identification of Tomato Golden 2-Like transcription factors and abiotic stress related members screening
Source: BMC Plant Biol. 2022 Feb 23;22:82. doi: 10.1186/s12870-022-03460-9 (PMC8864820; doi:10.1186/s12870-022-03460-9)

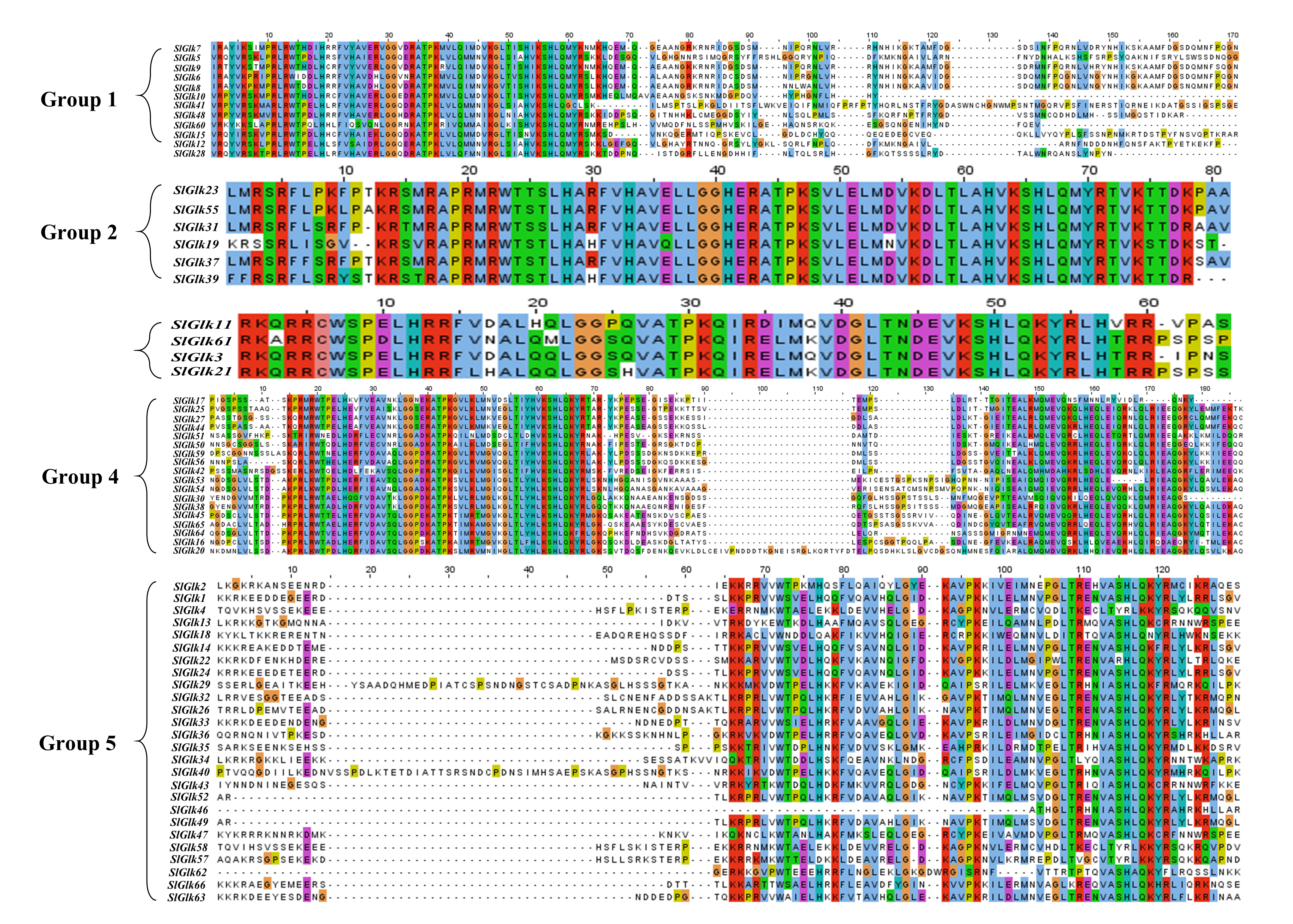

Supplement: Supplementary file 10 — Additional file 10. [file 12870_2022_3460_MOESM10_ESM.tif]

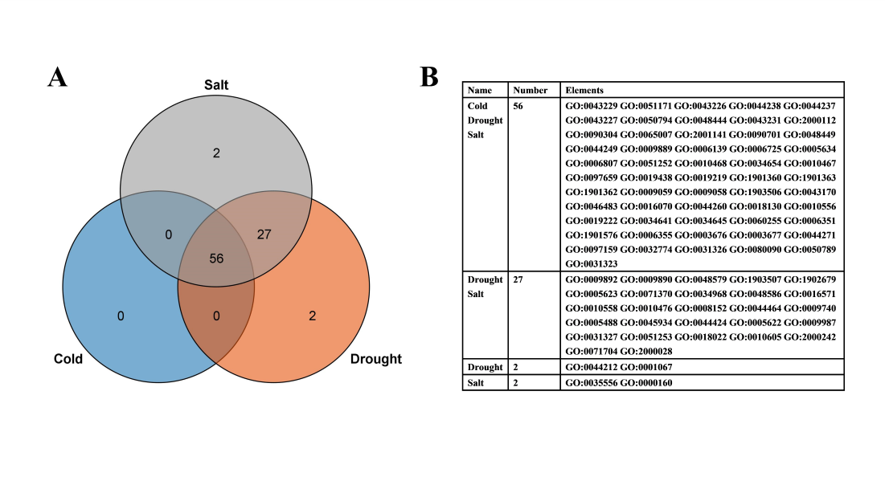

Supplement: Supplementary file 11 — Additional file 11. [file 12870_2022_3460_MOESM11_ESM.tif]

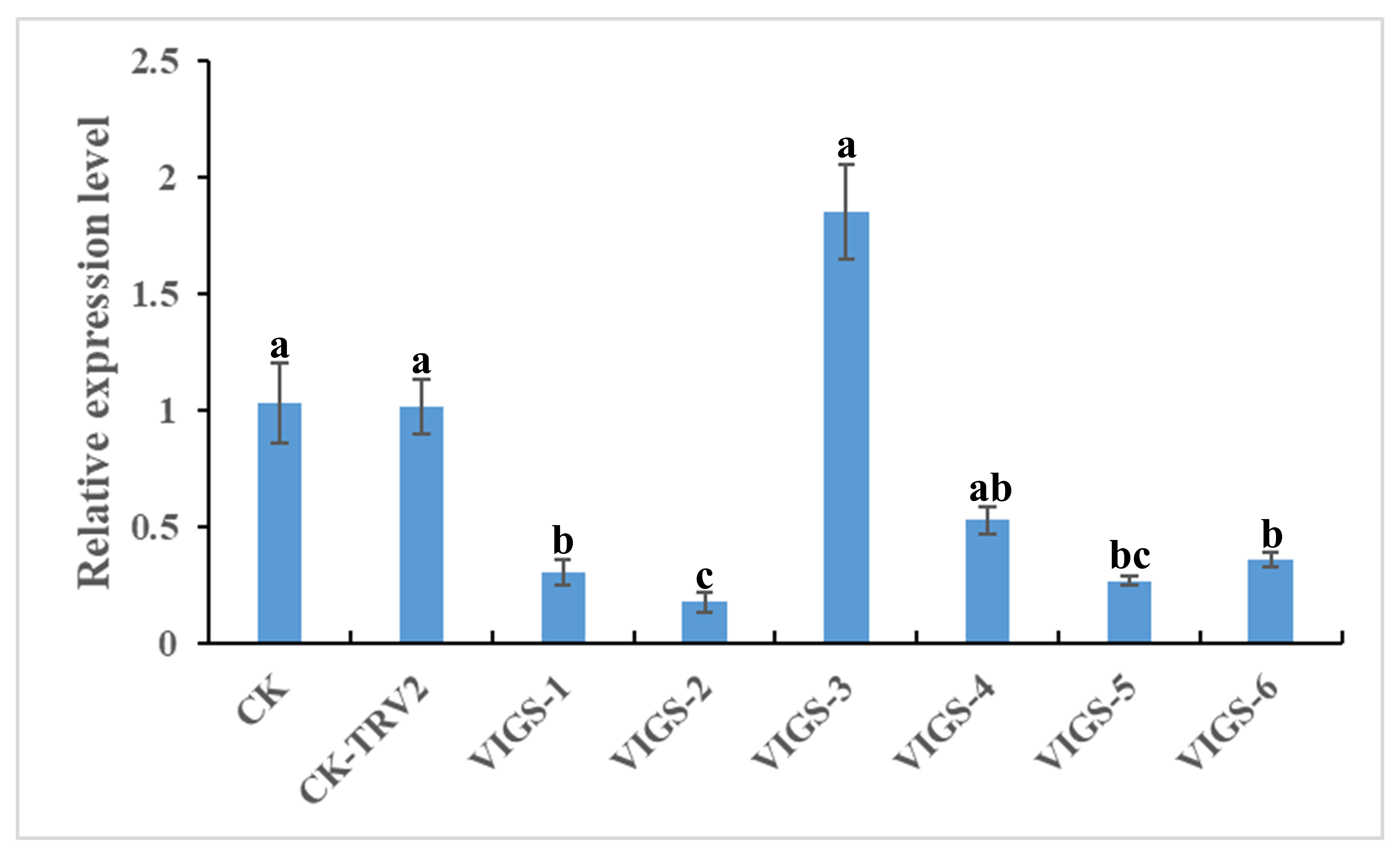

Supplement: Supplementary file 12 — Additional file 12. [file 12870_2022_3460_MOESM12_ESM.tif]
